# Supplementary material for: Proteomic sensors for quantitative multiplexed and spatial monitoring of kinase signaling
Source: Nat Commun. 2025 Nov 13;16:9902. doi: 10.1038/s41467-025-65950-2 (PMC12615678; doi:10.1038/s41467-025-65950-2)
Supplement: Supplementary file 1 — Supplementary Information [file 41467_2025_65950_MOESM1_ESM.pdf]

Supplementary Figures

A

Candidate	Sequence	Unphos Detected	Phos Detected
ATM c1	FEREDSQEEF*AGAR*	*Yes*	*Yes*
ATM c2	FEEDDSQEFY*AGAR*	*Yes*	*Yes*
ATM c3	YEDSDSQELE*AGAR*	*Yes*	*Yes*
ATM c4	FERSNSQDIW*AGAR*	*Yes*	*Yes*
ATM c5	YDDDDSQEFW*AGAR*	*Yes*	*Yes*
ATM c6	SERSFSQDLE*AGAR*	*Yes*	*Yes*
ATM c7	DEYEDSQEFY*AGAR*	*Yes*	*Yes*
ATM c8	FGREDSQEEI*AGAR*	*Yes*	*Yes*
ATM c9	WEESNSQDIW*AGAR*	*Yes*	*Yes*
ATM c10	FFDQDSQELE*AGAR*	*Yes*	*Yes*

B

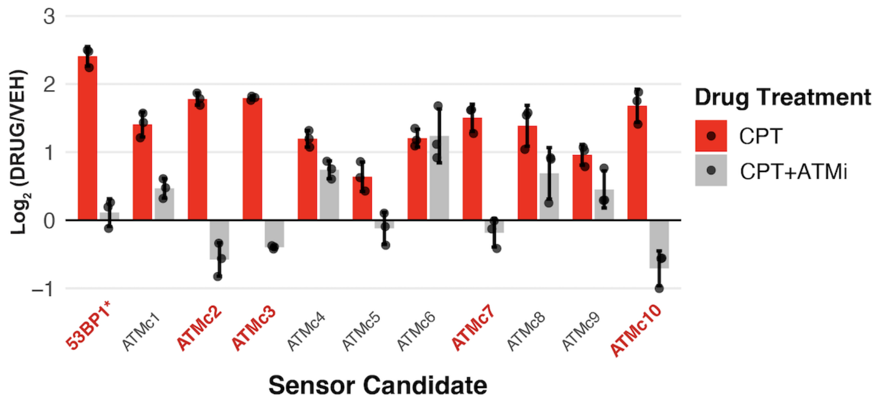

Supplementary Figure 1: Comparing phosphoproteome-derived sensors to sensors designed in silico

A) 10 ATM sensor candidates were generated *in silico* and cloned into a ProKAS biosensor for validation. Cells expressing this biosensor were treated with 1 micromolar CPT for 1 hour, and DDA MS analysis detected the phosphorylated form of all 10 candidates, confirming that the peptides ionize well and are phosphorylated *in vivo*.

B) The *in silico* ATM sensor candidates were co-expressed with the phosphoproteome-derived ATM sensor and phosphorylation levels were compared after 1 hour treatment with 1 micromolar CPT, 50 nanomolar ATMi (AZD-0156), or drug vehicle. *In silico* candidates with especially promising levels of induction and specificity are denoted in bold.

Error bars in B indicate the mean and standard deviation of triplicate independent experiments. Source data are provided as a Source Data file.

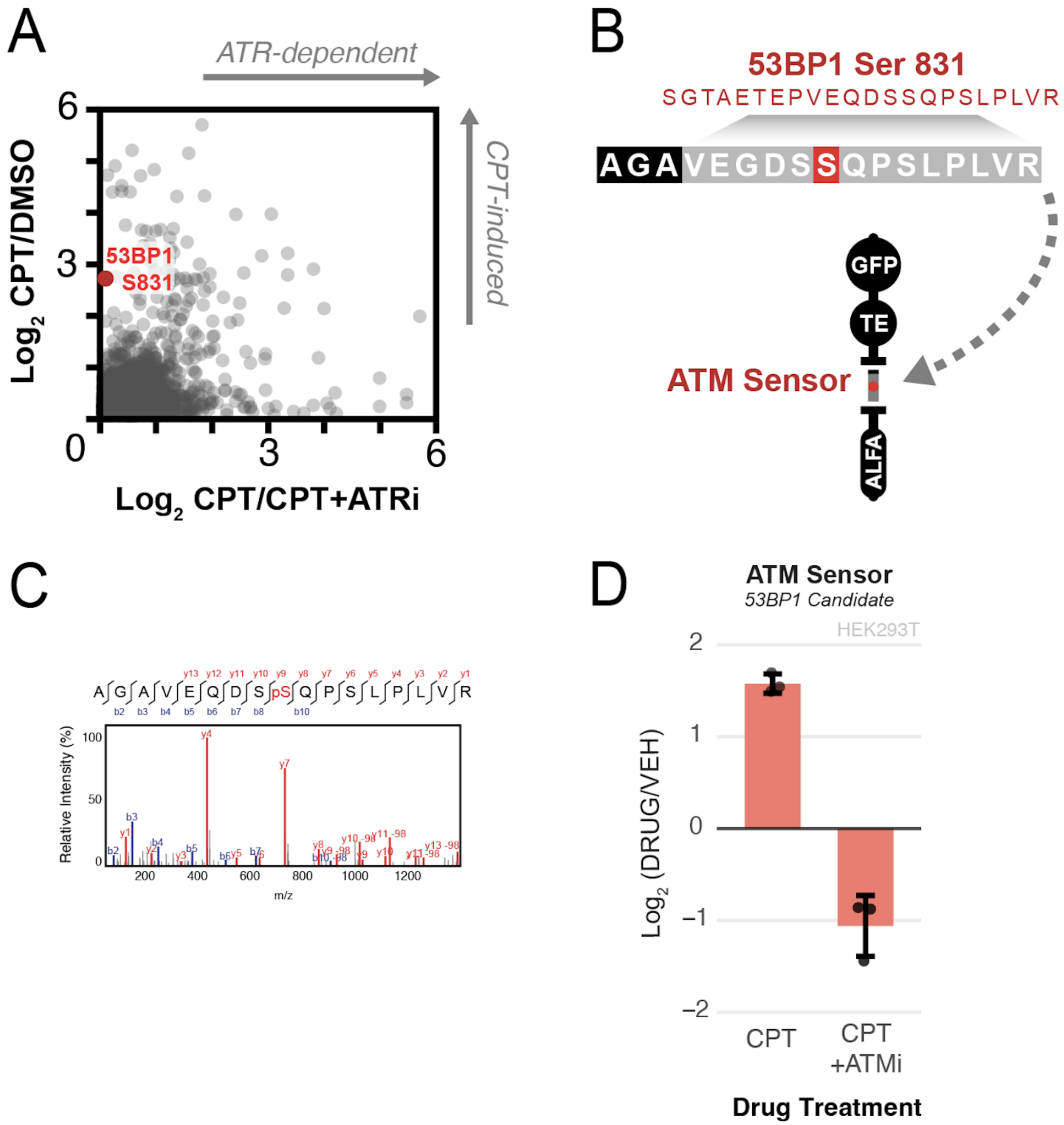

Supplementary Figure 2: Process for ATM sensor curation.

A) Phosphorylation site Serine 831 on 53BP1 was selected from the phosphoproteomic data generated to determine an ATR sensor. This site was induced by CPT but not dependent on ATR, and had previously been found to be phosphorylated by ATM. Phosphoproteome results included as Supplementary Data 3 and 4.

B) The 53BP1 Serine 831 phosphopeptide sequence was cloned into a ProKAS biosensor after adding a code to the N-terminal end.

C) Mass spectrometry was able to detect the phosphorylated form of this ATM sensor candidate, as shown by the MS/MS spectrum.

D) MS analysis showing inducibility and specificity of the ATM sensor candidate derived from 53BP1 after treatment with genotoxin and selective ATM inhibition, respectively. Cells were treated with 1 micromolar CPT for 30 minutes, and ATM-inhibited cells were treated with 50 nanomolar AZD-0156 15 minutes prior to CPT addition.

Error bars in D indicate the mean and standard deviation of triplicate independent experiments. Source data are provided as a Source Data file.

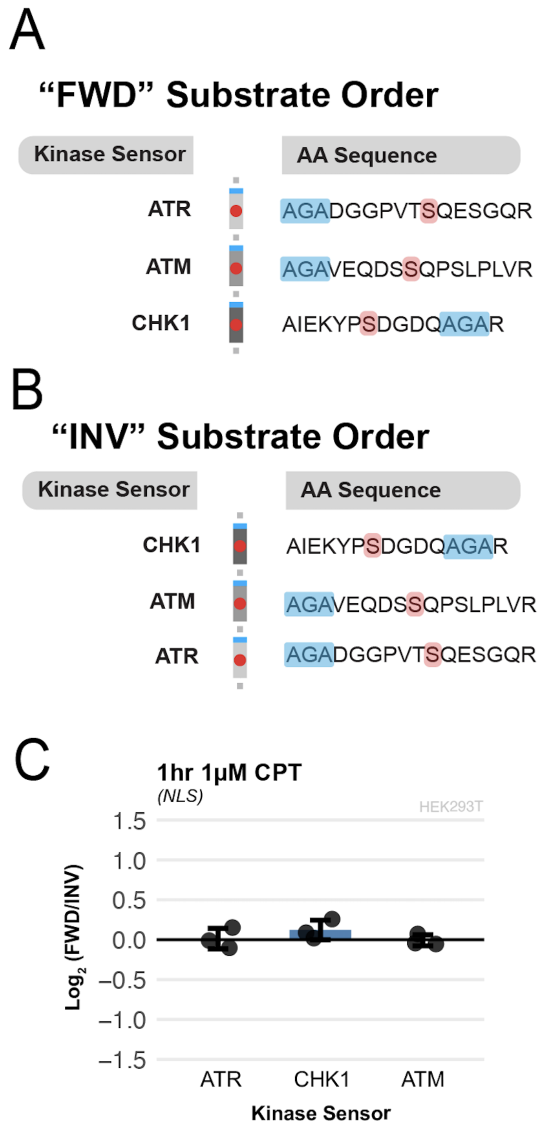

Supplementary Figure 3: ProKAS biosensor designs for testing the effect of kinase sensor order on phosphorylation levels.

A) The "forward" order of the kinase sensors: ATR - ATM - CHK1.

B) The "inverted" order of the kinase sensors: CHK1 - ATM - ATR.

C) Forward and inverted ProKAS biosensors showed no difference in sensor phosphorylation after 1 hour of treatment with 1 micromolar CPT.

Error bars in C indicate the mean and standard deviation of triplicate independent experiments. Source data are provided as a Source Data file.

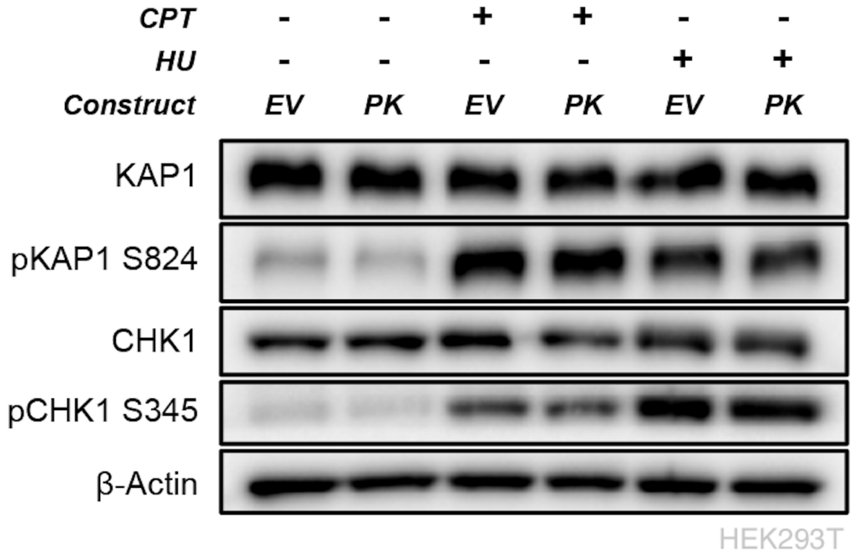

Supplementary Figure 4: Kinase sensor expression does not impact endogenous DDR signaling

Western blot where markers for ATM (KAP1 phosphorylation at S824) and ATR (CHK1 phosphorylation at S345) were measured in cells expressing an “EV” version of the ProKAS biosensor or the version containing kinase sensors for ATR, ATM, and CHK1 (denoted as “PK”). “EV” biosensors include all components of the ProKAS biosensor except for the MKS. Cells were treated with 1 micromolar CPT or 1 millimolar HU for 2 hours before lysis and Western blotting. Source data are provided as a Source Data file. Source data are provided as a Source Data file.

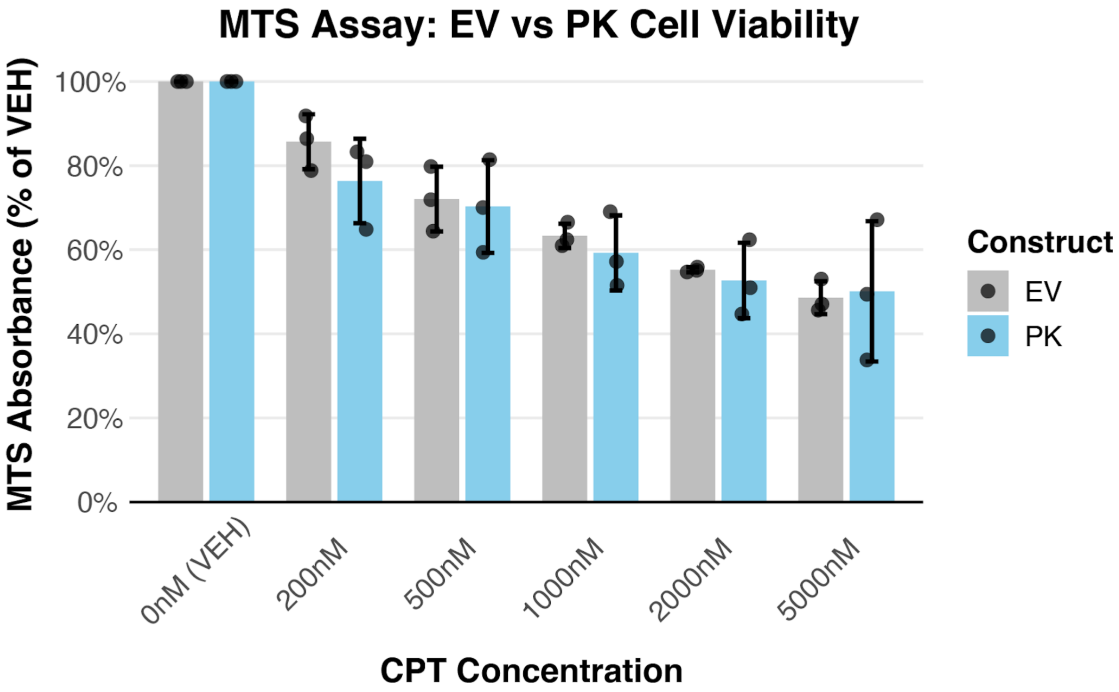

Supplementary Figure 5: Kinase sensor expression does not impact cell viability measured by MTS assay

MTS assay performed on cells reverse transfected with either the EV or PK versions of the biosensors as described in supplementary figure 4. 24 hours after reverse transfection, cells were treated with CPT at 200, 500, 1000, 2000, and 5000 nanomolar for 40 hours. MTS assay revealed no significant difference in cell viability between the EV- and PK-expressing cells after CPT treatment. Error bars indicate the mean and standard deviation of triplicate independent experiments. Source data are provided as a Source Data file.

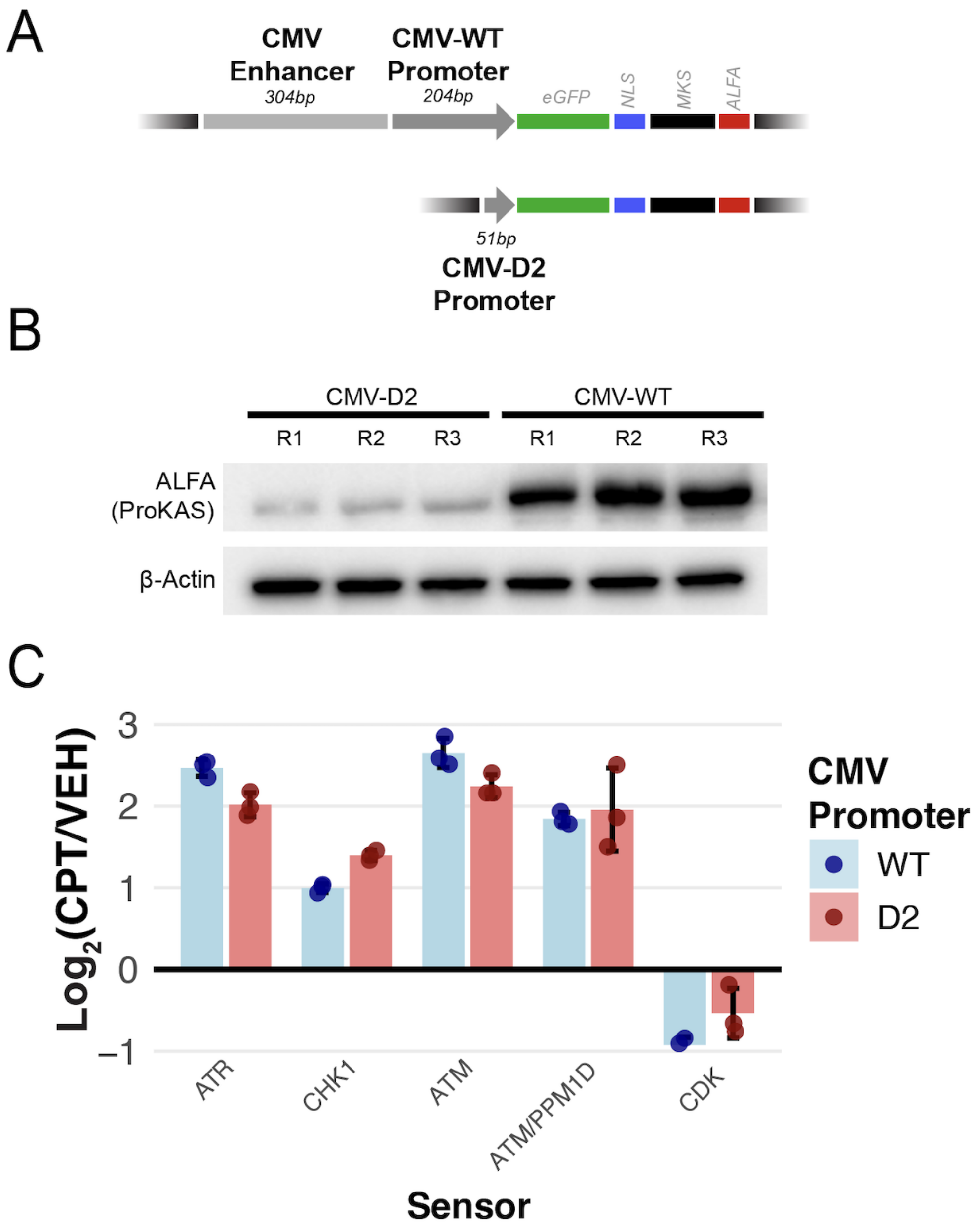

Supplementary Figure 6: Level of kinase sensor expression does not have a major impact on inducibility

A) Schematic illustrating the wild type (WT) and truncated (D2) versions of the CMV promoter used to compare expression levels of the ProKAS biosensor.

B) Western blot showing in triplicate that the truncated CMV promoter features far lower expression levels of the ProKAS biosensor (detected by blotting against the ALFA tag) than when using the wild type CMV promoter.

C) Quantification of kinase sensor peptide phosphorylation after expressing the ProKAS biosensor with either the D2 or WT CMV promoters and treating cells with 1 micromolar CPT for 2 hours.

Error bars in C indicate the mean and standard deviation of triplicate independent experiments. Source data are provided as a Source Data file.

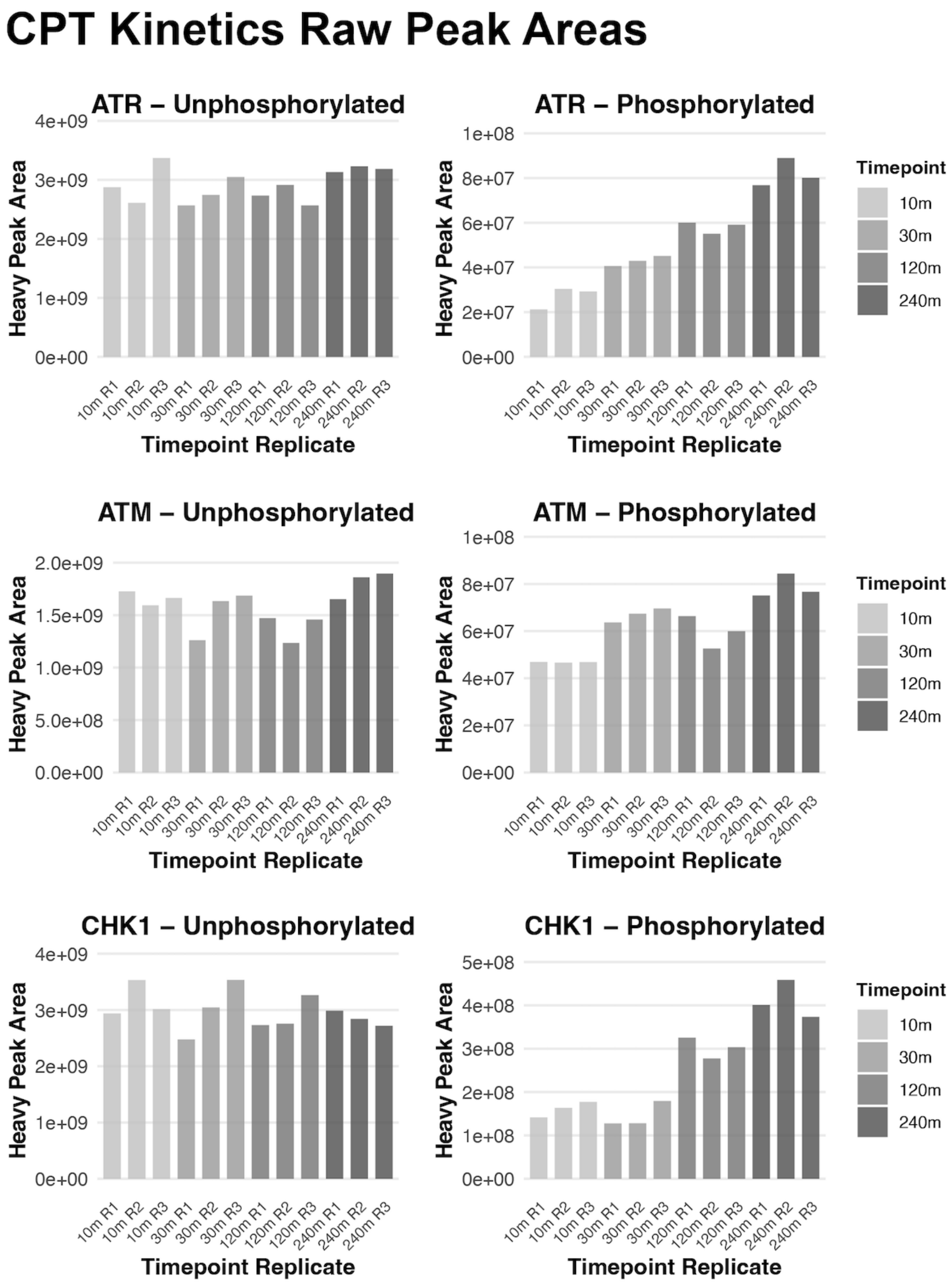

Supplementary Figure 7: Raw peak areas for CPT Kinetics

Raw heavy peak areas for all three sensor peptides quantified in figure 4E. Peak areas are displayed for both the unphosphorylated and phosphorylated versions of the peptides. The peak areas for each replicate are displayed discretely. Source data are provided as a Source Data file.

HU Kinetics Raw Peak Areas

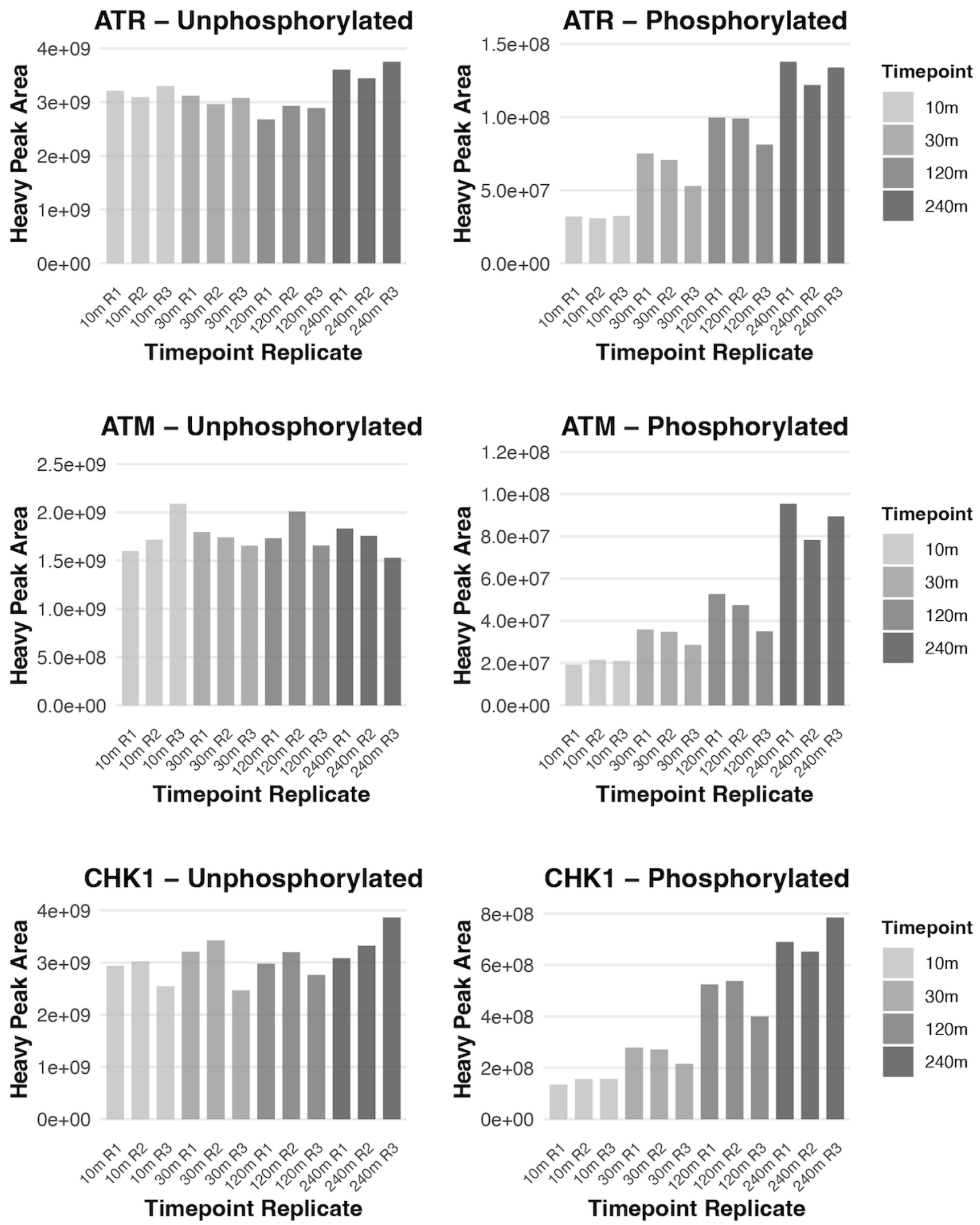

Supplementary Figure 8: Raw peak areas for HU Kinetics

Raw heavy peak areas for all three sensor peptides quantified in figure 4F. Peak areas are displayed for both the unphosphorylated and phosphorylated versions of the peptides. The peak areas for each replicate are displayed discretely. Source data are provided as a Source Data file.

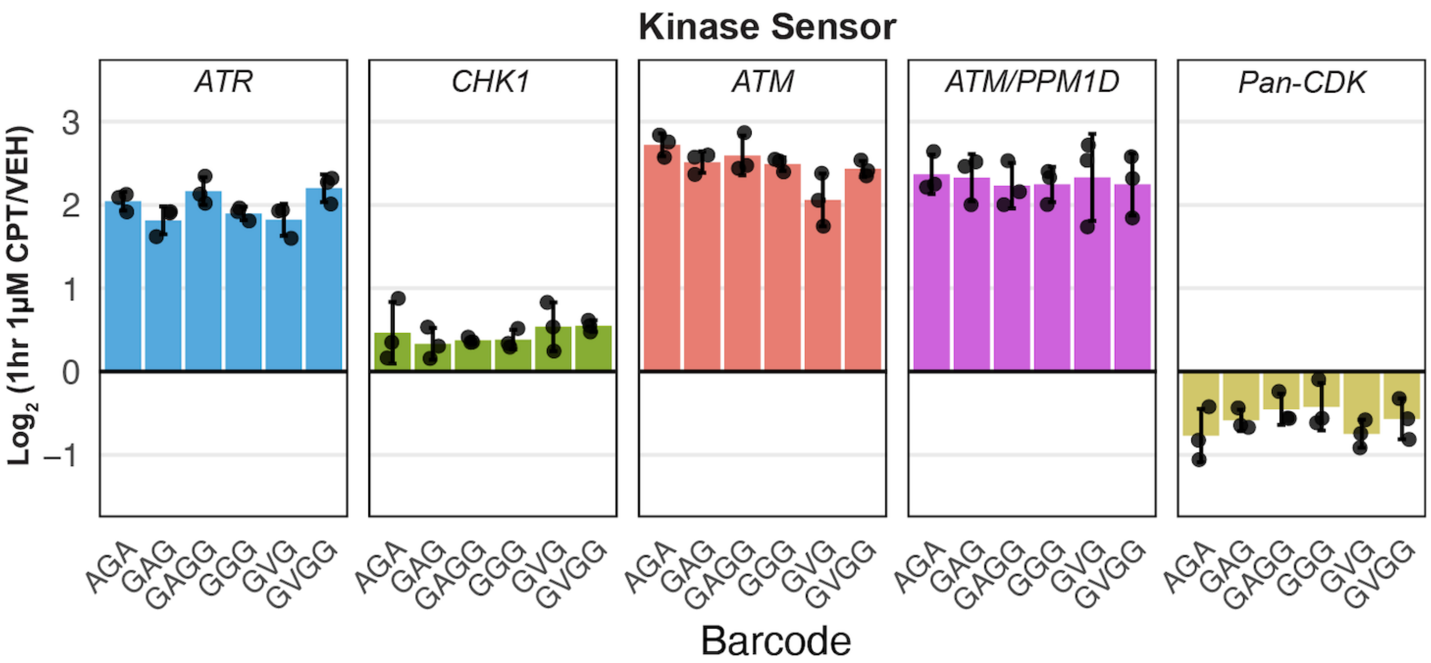

Supplementary Figure 9: Amino acid barcodes show no difference in phosphorylation efficiency.

Nuclear ProKAS constructs used in figure 6 with all 6 different amino acid barcodes (AGA, GAG, GAGG, GGG, GVG, and GVGG) were co-expressed in HEK293T cells and showed no significant difference in levels of induced phosphorylation after treatment with 1 micromolar CPT for 1 hour. Error bars/envelopes indicate the mean and standard deviation of triplicate independent experiments. Source data are provided as a Source Data file.

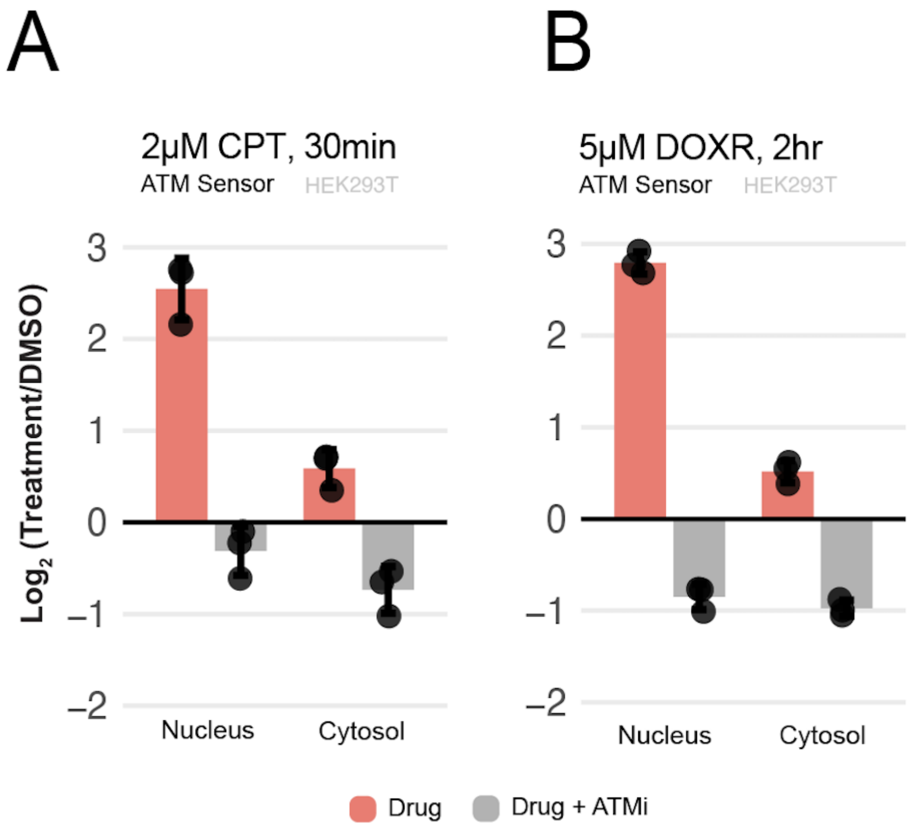

Supplementary Figure 10: Inhibition of nuclear and cytosolic ATM activity after CPT and DOXR treatment

A) ATM inhibitor removes induced phosphorylation of both the nuclear and cytosolic ATM sensor after treatment with 2 micromolar CPT for 30 minutes.

B) ATM inhibitor removes induced phosphorylation of both the nuclear and cytosolic ATM sensor after treatment with 5 micromolar DOXR for 2 hours.

Error bars in A and B indicate the mean and standard deviation of triplicate independent experiments. Source data are provided as a Source Data file.

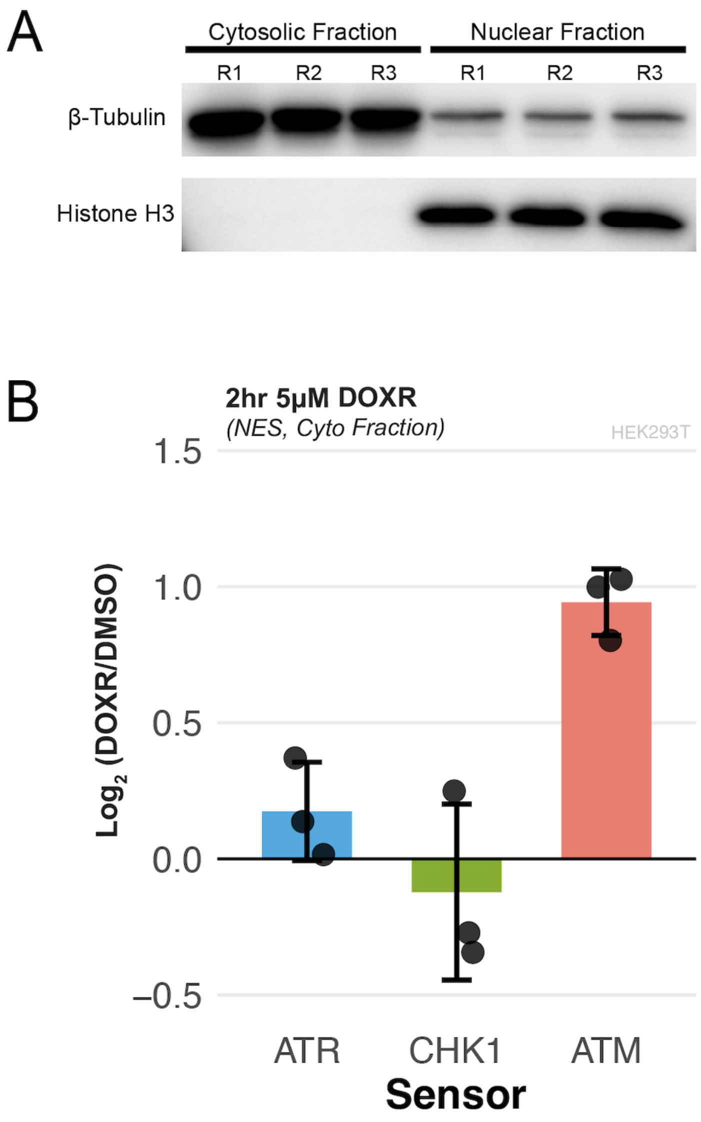

Supplementary Figure 11: ATM sensor phosphorylation is quantified in the cytosolic fraction

A) Western blot for β-tubulin and histone H3 in the cytosolic and nuclear fractions of cells expressing the cytosolic ProKAS biosensor used in figure 5. Source data are provided as a Source Data file.

B) Quantification of NES-containing ProKAS biosensors after affinity purification from the cytosolic fraction. Cells were treated with 5 micromolar DOXR for 2 hours prior to fractionation.

Error bars in B indicate the mean and standard deviation of triplicate independent experiments. Source data are provided as a Source Data file.

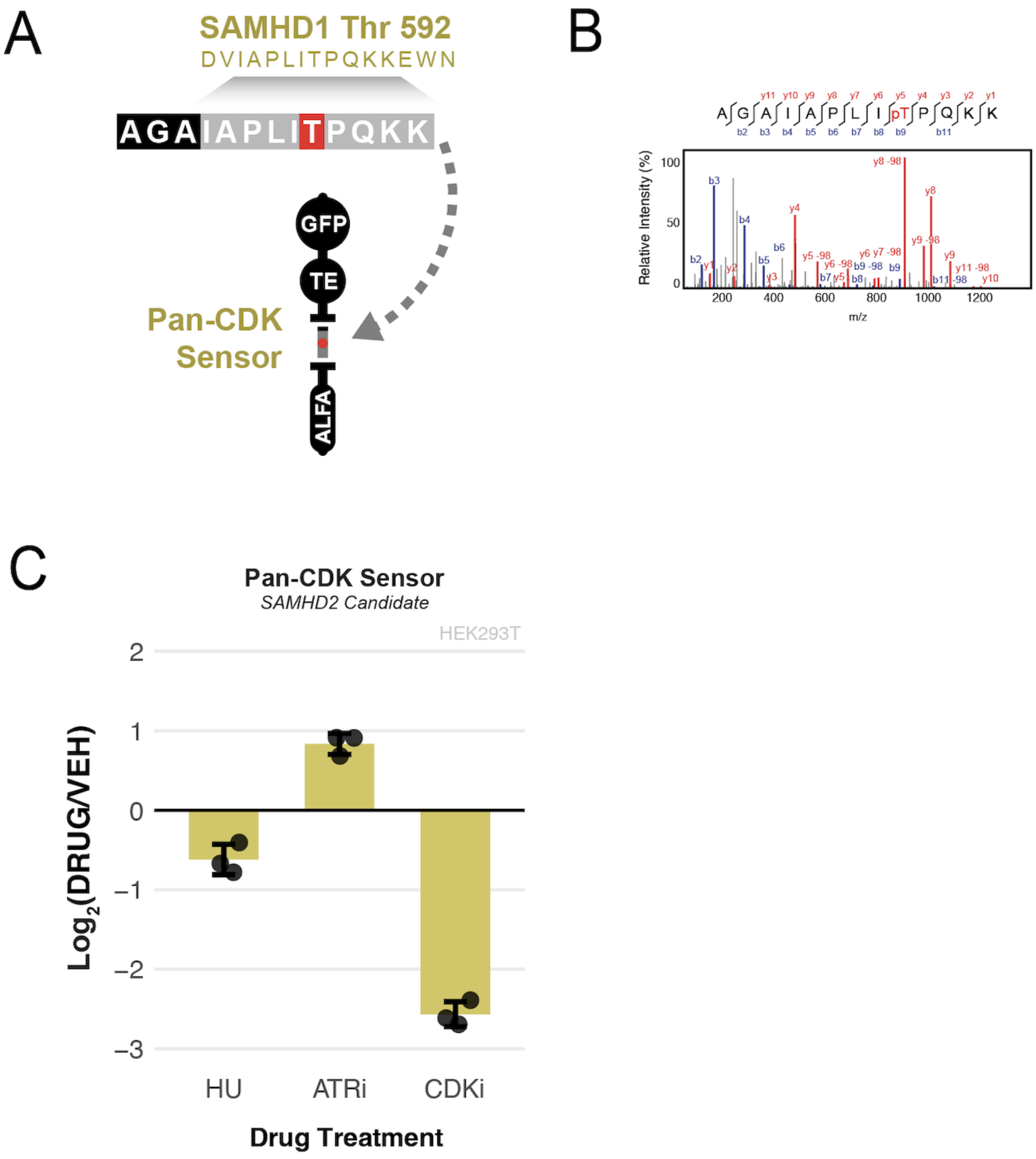

Supplementary Figure 12: Process for pan-CDK sensor curation

A) The SAMHD1 Threonine 592 phosphopeptide sequence was cloned into a ProKAS biosensor after adding a code to the N-terminal end.

B) Mass spectrometry was able to detect the phosphorylated form of this pan-CDK sensor candidate, as shown by the MS/MS spectrum.

C) MS analysis showing pan-CDK sensor phosphorylation reduced by treatment with HU, induced by ATR inhibition, and dramatically reduced by pan-CDK inhibitor, respectively. Cells were treated for 2 hours with 1 millimolar HU, 5 micromolar AZD-6738, or 5 micromolar flavopiridol.

Error bars in C indicate the mean and standard deviation of triplicate independent experiments. Source data are provided as a Source Data file.

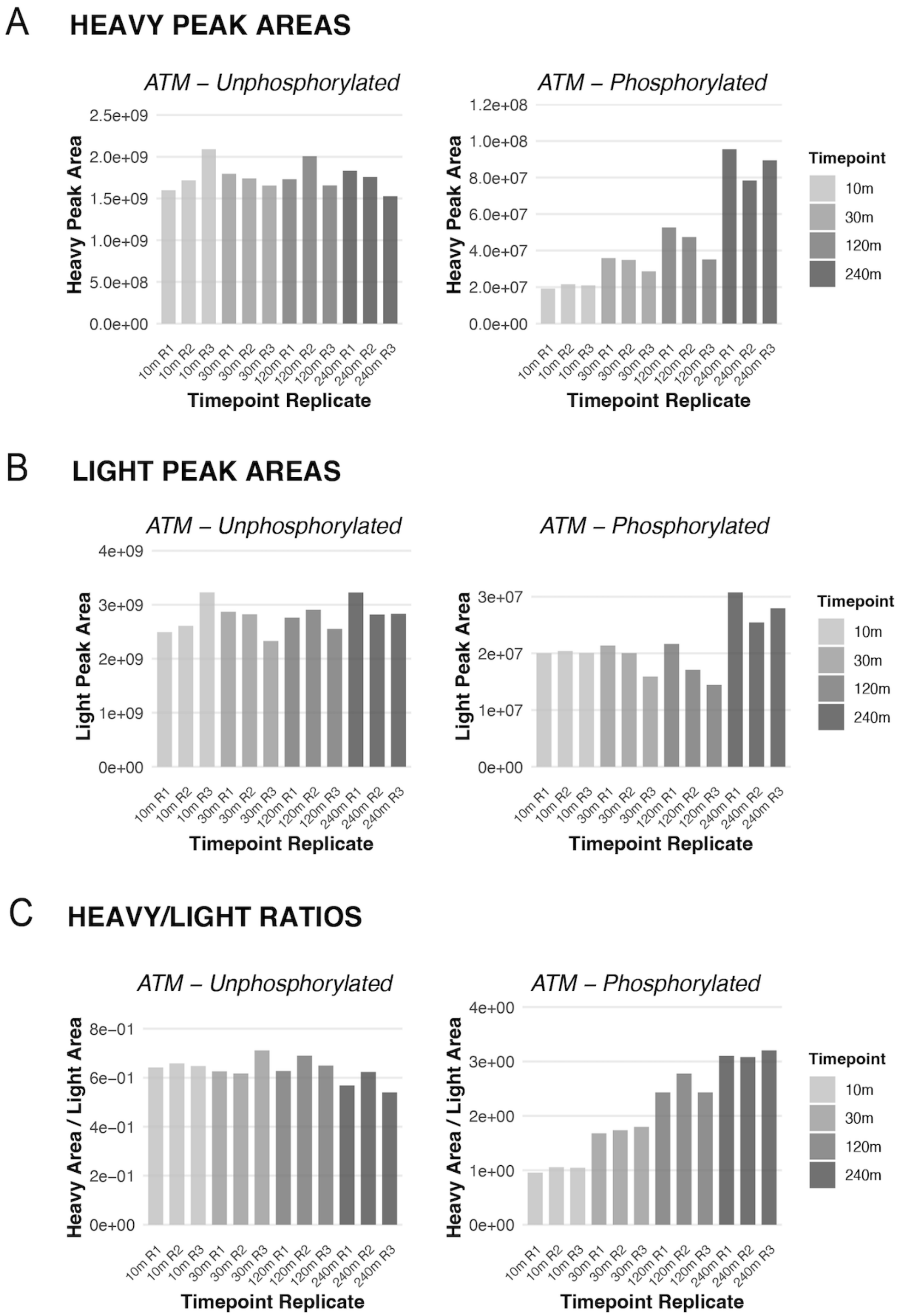

Supplementary Figure 13: Peak areas and SILAC ratios for nuclear ATM sensor

A) The raw heavy peak areas acquired for the nuclear ATM sensor at every timepoint in the HU kinetics displayed in figure 5D.

B) The raw light peak areas acquired for the nuclear ATM sensor at every timepoint in the HU kinetics displayed in figure 5D.

The Heavy/Light peak area ratios acquired for the nuclear ATM sensor at every timepoint in the HU kinetics displayed in figure 5D. Source data are provided as a Source Data file.
