# Supplementary material for: Proteomic sensors for quantitative multiplexed and spatial monitoring of kinase signaling
Source: Nat Commun. 2025 Nov 13;16:9902. doi: 10.1038/s41467-025-65950-2 (PMC12615678; doi:10.1038/s41467-025-65950-2)
Supplement: Supplementary file 2 — Description of Additional Supplementary Files [file 41467_2025_65950_MOESM2_ESM.pdf]

Description of Additional Supplementary Files

File Name: Supplementary Data 1.

Description: DNA sequences coding for targeting elements used in this study. Codon optimized for human expression.

File Name: Supplementary Data 2.

Description: DNA sequences coding for multiplexed kinase sensors (MKS) used in this study. Codon optimized for human expression.

File Name: Supplementary Data 3.

Description: Results of a phosphoproteomic screen looking for CPT-induced phosphorylation sites in HCT116 cells. Bowtie filtered. Sites in Q1 are CPT-induced, whereas sites in Q3 are CPT-reduced. Sites in CENTER are unchanged by CPT treatment.

File Name: Supplementary Data 4.

Description: Results of a phosphoproteomic screen looking for ATR-dependent phosphorylation sites induced by CPT in HCT116 cells. Sites with low Median Ratios are ATR-dependent.

File Name: Supplementary Data 5.

Description: Summary table of initial DDA analysis for 10 de novo CHK1 sensor candidates, of which 2 were found to be phosphorylated. Raw data is available upon request.

File Name: Supplementary Data 6.

Description: Summary table of the quantitative methods used for each ProKAS experiment displayed.

File Name: Supplementary Data 7.

Description: Raw MTS assay data used to create Supplementary Figure 5.

File Name: Supplementary Data 8

Description: Method files for mass spectrometer and KingFisher instrumentation.

File Name: Supplementary Data 9

Description: Full plasmid sequences for plasmids used throughout this study in .fasta format.

File Name: Supplementary Data 10

Description: Skyline document with transition and peptide settings configured for ProKAS analyses.

File Name: Supplementary Data 11

Description: TMT reagent isotope impurity information for quantification of TMT data acquired using Libra (as part of the Trans Proteomic Pipeline).

File Name: Supplementary Code 1

Description: Python script and accompanying files for generating kinase sensors candidate sequences de novo.
